# Inflammatory Diseases, Inflammatory Biomarkers, and Alzheimer Disease: An Observational Analysis and Mendelian Randomization

**DOI:** 10.1212/WNL.0000000000201489

**Published:** 2023-02-07

**Authors:** Jian Huang, Bowen Su, Ville Karhunen, Dipender Gill, Verena Zuber, Ari Ahola-Olli, Saranya Palaniswamy, Juha Auvinen, Karl-Heinz Herzig, Sirkka Keinänen-Kiukaanniemi, Marko Salmi, Sirpa Jalkanen, Terho Lehtimäki, Veikko Salomaa, Olli T. Raitakari, Paul M. Matthews, Paul Elliott, Konstantinos K. Tsilidis, Marjo-riitta Jarvelin, Ioanna Tzoulaki, Abbas Dehghan

**Affiliations:** From the Department of Epidemiology and Biostatistics (J.H., B.S., V.K., D.G., V.Z., S.P., P.E., K.K.T., M.-r.J., A.D.), School of Public Health, Imperial College London, United Kingdom; Singapore Institute for Clinical Sciences (SICS) (J.H.), Agency for Science, Technology and Research (A*STAR); Center for Life Course Health Research (V.K., S.P., J.A., S.K.-K., M.-r.J.), Faculty of Medicine, Research Unit of Mathematical Sciences (V.K.), University of Oulu, Finland; The Stanley Center for Psychiatric Research (A.A.-O.), Broad Institute of MIT and Harvard, Cambridge, MA; Analytical and Translational Genetics Unit (A.A.-O.), Massachusetts General Hospital, Boston; Institute for Molecular Medicine Finland (A.A.-O.), University of Helsinki; Research Unit of Biomedicine (K.-H.H.), Medical Research Center (MRC), University of Oulu, University Hospital, Finland; Department of Gastroenterology and Metabolism (K.-H.H.), Poznan University of Medical Sciences, Poland; Unit of Primary Care (S.K.-K., M.-r.J.), Oulu University Hospital; Healthcare and Social Services of Selänne (S.K.-K., I.T.), Pyhäjärvi, Finland and City of Oulu; MediCity and Institute of Biomedicine (M.S., S.J.), University of Turku; Department of Clinical Chemistry (T.L.), Fimlab Laboratories, and Finnish Cardiovascular Research Center, Tampere, Faculty of Medicine and Health Technology, Tampere University; Finnish Institute for Health and Welfare (V.S.), Helsinki; Research Centre of Applied and Preventive Cardiovascular Medicine (O.T.R.), University of Turku; Department of Clinical Physiology and Nuclear Medicine (O.T.R.), Turku University Hospital; Centre for Population Health Research (O.T.R.), University of Turku and Turku University Hospital, Finland; Department of Brain Sciences (P.M.M.), Faculty of Medicine, Imperial College London; UK Dementia Research Institute at Imperial College London (P.M.M., P.E.); MRC Centre for Environment and Health (P.E., M.-r.J.), School of Public Health, Imperial College London, United Kingdom; Department of Hygiene and Epidemiology (K.K.T.), University of Ioannina Medical School, Greece; Biocenter Oulu (M.-r.J.), University of Oulu, Finland; and Department of Life Sciences (M.-r.J.), College of Health and Life Sciences, Brunel University London, United Kingdom.

## Abstract

**Background and Objectives:**

Whether chronic autoimmune inflammatory diseases causally affect the risk of Alzheimer disease (AD) is controversial. We characterized the relationship between inflammatory diseases and risk of AD and explored the role of circulating inflammatory biomarkers in the relationships between inflammatory diseases and AD.

**Methods:**

We performed observational analyses for chronic autoimmune inflammatory diseases and risk of AD using data from 2,047,513 participants identified in the UK Clinical Practice Research Datalink (CPRD). Using data of a total of more than 1,100,000 individuals from 15 large-scale genome-wide association study data sets, we performed 2-sample Mendelian randomizations (MRs) to investigate the relationships between chronic autoimmune inflammatory diseases, circulating inflammatory biomarker levels, and risk of AD.

**Results:**

Cox regression models using CPRD data showed that the overall incidence of AD was higher among patients with inflammatory bowel disease (hazard ratio [HR] 1.17; 95% CI 1.15–1.19; *p* = 2.1 × 10^−4^), other inflammatory polyarthropathies and systematic connective tissue disorders (HR 1.13; 95% CI 1.12–1.14; *p* = 8.6 × 10^−5^), psoriasis (HR 1.13; 95% CI 1.10–1.16; *p* = 2.6 × 10^−4^), rheumatoid arthritis (HR 1.08; 95% CI 1.06–1.11; *p* = 4.0 × 10^−4^), and multiple sclerosis (HR 1.06; 95% CI 1.04–1.07; *p* = 2.8 × 10^−4^) compared with the age (±5 years) and sex-matched comparison groups free from all inflammatory diseases under investigation. Bidirectional MR analysis identified relationships between chronic autoimmune inflammatory diseases and circulating inflammatory biomarkers. Particularly, circulating monokine induced by gamma interferon (MIG) level was suggestively associated with a higher risk of AD (odds ratio from inverse variance weighted [OR_IVW_] 1.23; 95% CI 1.06–1.42; *p*_IVW_ = 0.007) and lower risk of Crohn disease (OR_IVW_ 0.73; 95% CI −0.62 to 0.86; *p*_IVW_ = 1.3 × 10^−4^). Colocalization supported a common causal single nucleotide polymorphism for MIG and Crohn disease (posterior probability = 0.74), but not AD (posterior probability = 0.03). Using a 2-sample MR approach, genetically predicted risks of inflammatory diseases were not associated with higher AD risk.

**Discussion:**

Our data suggest that the association between inflammatory diseases and risk of AD is unlikely to be causal and may be a result of confounding. In support, although inflammatory biomarkers showed evidence for causal associations with inflammatory diseases, evidence was weak that they affected both inflammatory disease and AD.

Progressive cerebral neurodegeneration with extracellular β-amyloid (Aβ) plaques and intraneuronal neurofibrillary tangles are pathologic features distinguishing Alzheimer disease (AD), the most common cause of dementia. The brains of patients with AD show evidence for a sustained brain innate immune response.^1^ Moreover, higher levels of circulating inflammatory markers in the blood were observed in patients with AD compared with that of healthy controls.^2^ Observational studies and meta-analyses have reported a higher risk of dementia among patients with rheumatoid arthritis (RA),^3^ psoriasis,^4^ and inflammatory bowel disease (IBD),^5^ although contradictory results also have been reported.^6,7^ Whether chronic autoimmune inflammatory diseases causally affect the risk of AD is controversial.

The observed associations between these inflammatory diseases and AD may be because of common circulating inflammatory biomarkers, modulation of which could provide opportunities for the prevention and treatment of AD. However, previous studies showing inconsistent effects of anti-inflammatory drugs on AD have cast doubt on the potential for therapeutic modulation of chronic inflammation in AD.^8,9^ In this study, we investigated the relationship between different chronic autoimmune inflammatory diseases and risks of AD/dementia using a real-world observational analysis using large-scale population-based electronic health records (EHRs) and an instrumental variable analysis with genetic instruments known as Mendelian randomization (MR), which is less susceptible to confounding and reverse causation than conventional observational analysis.^10^ We performed bidirectional MR to further examine the role of circulating inflammatory proteins associated with or induced by autoimmune disease in the relationships between inflammatory diseases and AD. Identification of such inflammatory proteins could identify potential therapeutic targets for AD.

## Methods

### Real-World Observational Analysis

#### Clinical Practice Research Datalink

The study cohort was selected from the Clinical Practice Research Datalink (CPRD) between January 1, 1987, and May 3, 2018, with the primary care data linking to secondary care data from Hospital Episode Statistics (HES), mortality data from the Office of National Statistics (ONS), and small-area measures of social deprivation.^11^

#### Sampling

We designed a cohort study in which exposed participants were eligible CPRD participants who were diagnosed with one of the following inflammatory diseases: (1) RA, (2) IBD, (3) multiple sclerosis (MS), (4) psoriasis, and (5) other inflammatory polyarthropathies and systematic connective tissue disorders (OID) (eMethods, links.lww.com/WNL/C487 and eTables 1–5, links.lww.com/WNL/C486). Exposed participants were excluded if their dates of inflammatory disease diagnoses were missing or if their dates of inflammatory disease diagnoses were after or less than 1 year before their incident AD diagnosis. Each eligible exposed participant was matched on age (±5 years) and sex with 2 nonexposed participants who had no record of these inflammatory diseases from the remaining CPRD participants.

#### Outcome Definition

AD case was defined by the presence of 1 or more AD diagnosis codes from a selected list of CPRD Medcodes or *International Classification of Diseases, 10th Revision* codes in linked HES or ONS data sets (eTable 6, links.lww.com/WNL/C486). The AD diagnosis date was defined as the date of the first AD recording in the CPRD, HES, or ONS data set. Where dates of diagnoses were missing, the diagnosis records were excluded. We identified 134,952 AD cases for our analysis (eFigure 1, links.lww.com/WNL/C487).

#### Conventional Cox Regression Models and Propensity Score Analysis

Conventional Cox regression models with follow-up time as the underlying timescale were used to estimate the risk of developing AD with hazard ratios (HRs) and 95% CIs with the presence of inflammatory disease. Eligible exposed participants were coded as 1 while nonexposed participants were coded as 0. The time of the first record of inflammatory disease diagnosis for exposed participants and their matched nonexposed participants was considered the baseline of the cohort. The end of follow-up was defined as the date of (1) AD incidence (as defined above), (2) death, (3) practice transfer out date, (4) last data collection date of general practitioner (GP) practice, or (5) last follow-up date from CPRD (May 1, 2018), whichever occurred first (eFigure 2, links.lww.com/WNL/C487).

Covariates that potentially influenced or strongly associated with the AD onset were adjusted for in Cox regression models. These included region in the United Kingdom; index of multiple deprivation (IMD, a proxy of socioeconomic status linked to CPRD); body mass index (BMI, latest record up to 5 years before baseline to reduce missing values); smoking status (latest record up to 5 years before baseline); number of GP consultations (number of records before baseline); and history of major comorbidities, including cardiovascular diseases (coronary heart disease, heart failure, stroke, peripheral arterial disease) and type II diabetes. For propensity score analysis, we adjusted for confounding by rebalancing each inflammatory disease and nonexposed groups, using inverse propensity score treatment weighting (IPTW) to account for selection assignment differences between inflammatory diseases and their comparison groups.^12^ For missingness of IMD, BMI, and smoking information, multiple imputations with chained equations were used in the Cox regression models and propensity score weighting models to address missingness.^13^

#### Competing Risk Analysis

Competing risk analysis using IPTW and adjusting for the same set of covariates as included in the Cox regression models was used to estimate the single-world cause-specific hazards and cumulative incidence functions (CIFs), which indicate the effect of inflammatory diseases on the risk of dementia in the presence of competing death.^14^

#### Sensitivity Analyses

We also performed sensitivity analyses to assess the robustness of the analyses: (1) restricting the analyses to eligible exposed participants with their first inflammatory disease diagnosis after 2004 because of the implementation of pay for performance in primary care and improvement of CPRD recording^15^; (2) restricting the analyses to participants who could be linked to HES or ONS data sets (linkage data sets), which provide additional information on inflammatory diseases, AD, or death recordings; (3) using the recording of dementia diagnosis as the outcome; (4) using patients with both an AD diagnosis in CPRD and HES/ONS plus at least 1 dementia drug prescription in CPRD; (5) removing AD cases that developed within 2 years of inflammatory disease diagnosis; and (6) removing AD cases that developed within 3 years of inflammatory disease diagnosis (eTable 6, links.lww.com/WNL/C486). See eMethods (links.lww.com/WNL/C487) for more details.

### Mendelian Randomization

#### Genetic Associations of Circulating Inflammatory Biomarkers

We selected circulating inflammatory biomarkers of which meta-analyses of a genome-wide association study (GWAS) were available based on 3 Finnish cohorts, namely Northern Finland Birth Cohort 1966, the Cardiovascular Risk in Young Finns, and FINRISK.^16^ To increase the power of our analysis, we further incorporated the Finnish meta-analysis with summary statistics from GWAS on proteins in the INTERVAL study^17^ and SCALLOP consortium.^18^ Details of the meta-analysis are described in eMethods (links.lww.com/WNL/C487) and eTable 7 (links.lww.com/WNL/C486).

#### Genetic Associations of Inflammatory Diseases, AD, and Dementia

We obtained genetic associations for 8 inflammatory diseases from the largest GWAS, including psoriasis, RA, MS, IBD, and the 2 subtypes of IBD (Crohn disease and ulcerative colitis [UC]). Case ascertainment of inflammatory diseases was based on clinical diagnosis recorded in the hospital or self-reported records. Genetic associations of risk of late-onset AD were obtained from the GWAS meta-analysis by the International Genomics of Alzheimer's Project (IGAP).^19^ The discovery stage of the IGAP GWAS meta-analysis included 21,982 cases (clinically diagnosed or autopsy) and 41,944 cognitively normal controls of European ancestry.^19^ As a sensitivity analysis, we also used genetic associations of dementia obtained from a meta-analysis of the IGAP, GR@ACE, and UK Biobank (N = 409,435 individuals of European ancestry).^20^ See eMethods (links.lww.com/WNL/C487) and eTable 8 (links.lww.com/WNL/C486) for more details.

#### Two-Sample MR

We performed 2-sample MR to investigate 3 sets of associations, that is, (1) the associations of genetic liability of inflammatory diseases with AD/dementia, (2) the associations of genetic liability of inflammatory diseases and AD with circulating inflammatory biomarker levels, and (3) the associations of genetically predicted circulating inflammatory biomarker levels with the risk of inflammatory diseases and AD. For each MR analysis, we selected genetic instruments with a *p* value smaller than 5 × 10^−8^ with the exposure of interest. We included independent single nucleotide polymorphisms (SNPs) as genetic instruments when genetic associations were available for both the exposure and outcome of interest. Correlated SNPs (*r*^2^ > 0.001) were excluded by keeping the one with the smallest *p* value for the SNP exposure association. To avoid weak instrument bias, we only included SNPs with an *F*-statistic greater than 10.^10^

In our primary method, we used the Wald ratio to estimate SNP-specific effects when only 1 instrument was available.^21^ For MR analyses with 2 or 3 instruments, we used an inverse variance weighted (IVW) fixed-effects model for the MR effect estimates.^21^ For MR analyses with more than 3 instruments, we used the IVW random-effects model for the MR effect estimates.^21^ We, in addition, used 2 sensitivity methods, weighted median and MR-Egger regression, to assess the robustness and horizontal pleiotropic effects when more than 2 instruments were available for the analysis.^21^ We also excluded potential outlier SNPs using MR-PRESSO, which identified outliers by comparing the observed and expected residual sum of squares for each SNP when regressing the SNP exposure association on the SNP outcome association.^22^ We performed a sensitivity analysis excluding genetic instruments on chromosome 6 for the association of inflammatory diseases with AD/dementia because of the complex linkage disequilibrium issue of the *HLA* genes. For analyses with biomarker level as the exposure, we reported the odds ratio (OR) for the disease outcome per SD higher of circulating inflammatory biomarker level. For the analyses with a disease exposure, the original result output indicates the effect on an outcome per unit increase in the ln(OR) of exposure risk. We multiplied the effect by 0.693 (i.e., ln(2)) to report the effect on a continuous outcome or the OR for a binary outcome per doubling in odds of the disease exposure.

We performed MR-Steiger to further explore the directionality for the biomarker-disease pairs showing a bidirectional relationship.^23^ MR-Steiger infers the directionality of the association by assessing the variance explained in the exposure and outcome by the genetic instruments.^23^ In addition, we performed sensitivity MR analyses excluding SNPs that were nominally associated with the outcome (*p* < 0.05) for the biomarker-disease pairs of interest.

We performed post hoc power analysis for the MR for the associations of genetically predicted circulating inflammatory biomarker levels with the risk of inflammatory diseases and AD.^24^ Specifically, we approximated the proportion of variance explained by the independent genetic variants for the circulating level of an inflammatory biomarker and estimated the minimum effect detectable for each association at an 85% power.

We accounted for multiple comparisons using Bonferroni correction. Specifically, in the MR analysis for the associations of genetically predicted inflammatory diseases with AD, we accounted for multiple comparisons of 6 inflammatory diseases using a *p* value threshold of 0.05/6 = 0.0083. In the bidirectional MR analysis for circulating inflammatory biomarker levels and diseases of interest, we accounted for multiple comparisons of 533 unique associations (322 for the effects of 7 diseases on 46 circulating inflammatory biomarkers and 211 for the effects of circulating inflammatory biomarkers on the diseases) with a *p* value threshold of 0.05/533 = 9.4 × 10^−5^.

#### Colocalization

We further performed colocalization testing for the genetic signals for AD and Crohn disease to investigate whether the 2 diseases shared causal variants. Specifically, we focused on the genomic region within 50 kb from the coding gene of monokine induced by gamma interferon (MIG) (*CXCL9*, 4:76922428–76928641) given the associations identified in the 2-sample MR analyses. We also performed colocalization for MIG with AD and MIG with Crohn disease. Given the functional relevance between MIG and IP10 (*CXCL10*, 4:76942273–76944650), we also performed colocalization for IP10 with AD and IP10 with Crohn disease.

### Statistical Software

All analyses were performed in Stata (version 16) and R 4.1.2. The *causalCmprsk* R package was used for the competing risk analysis.^14^ MR analyses were performed using the *TwoSampleMR* and *MR-PRESSO* packages. Colocalization was performed using the *coloc* package. All statistical tests were 2-sided.

### Standard Protocol Approvals, Registrations, and Patient Consents

The use of CPRD data for this study was approved by the Independent Scientific Advisory Committee for Medicines and Healthcare products Regulatory Agency database research (protocol number: 20_000209). Two-sample MR analyses were performed using publicly available GWAS; written informed consent was obtained from the participants by the individual cohort being involved in the GWAS.

### Data Availability

Access to CPRD data is subject to protocol approval by CPRD's Research Data Governance Process. GWAS data on inflammatory diseases, AD, and dementia are available for download through the corresponding publication. GWAS on proteins in the INTERVAL study and SCALLOP consortium is available for download through the corresponding publication. Summary statistics for the Finnish meta-analysis are available on request.

## Results

### Observational Analysis (CPRD)

**Figure 1:**
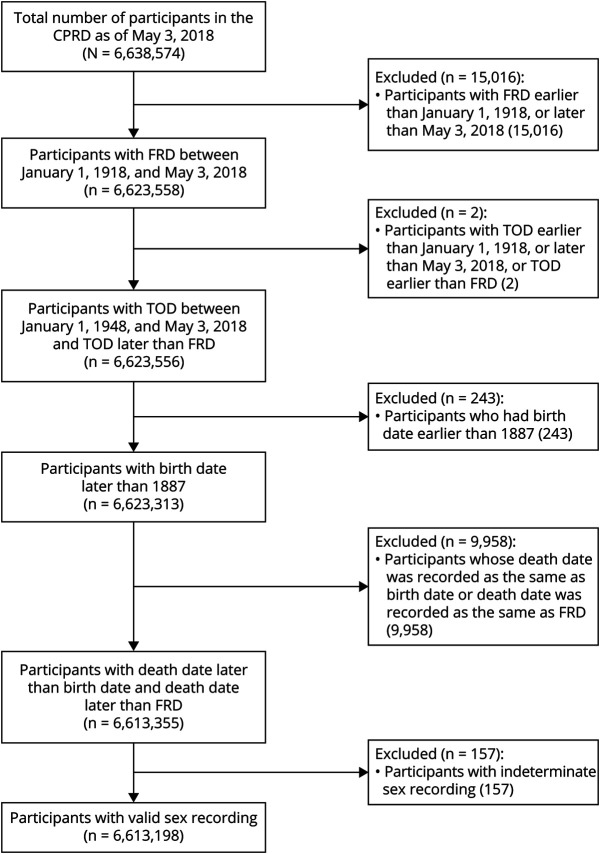
Flowchart of Inclusion and Exclusion Criteria of Study Population in the CPRD CPRD = Clinical Practice Research Datalink; FRD = first registration date; TOD = transfer out date.

The CPRD cohort included a total of 6,613,198 participants who met our quality checks (Figure 1). We included 85,147 patients with RA; 57,114 patients with IBD; 20,743 patients with MS; 220,729 patients with psoriasis; and 339,960 patients with OID in the analyses with a median clinical follow-up of 13 years. There were more obese patients and more patients with cardiovascular diseases and type 2 diabetes in patients diagnosed with inflammatory diseases, except for MS at baseline, than the nonexposed (Table 1).

**Table 1:**
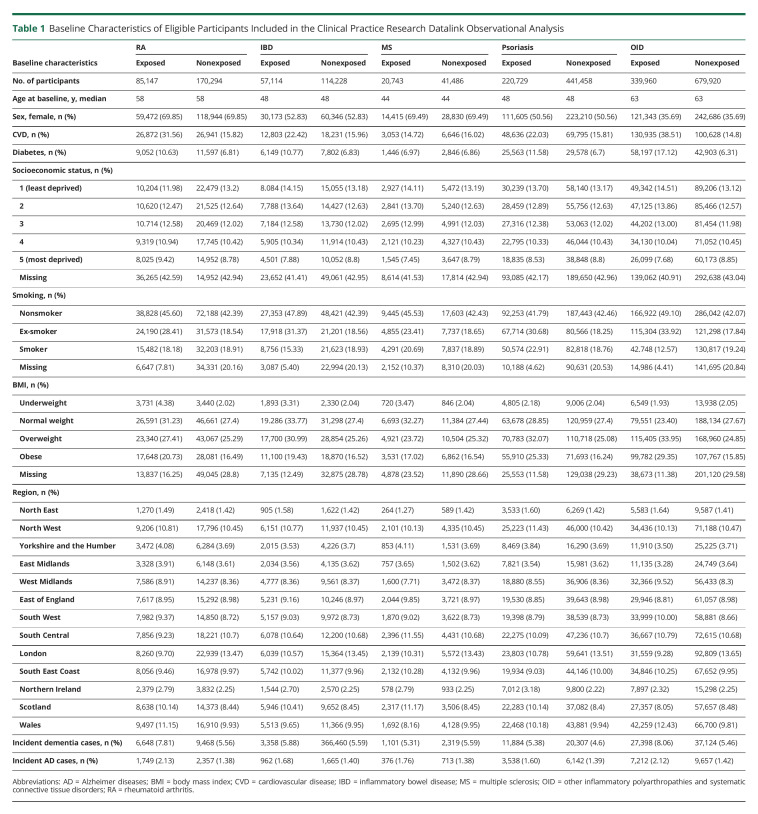
Baseline Characteristics of Eligible Participants Included in the Clinical Practice Research Datalink Observational Analysis

**Figure 2:**
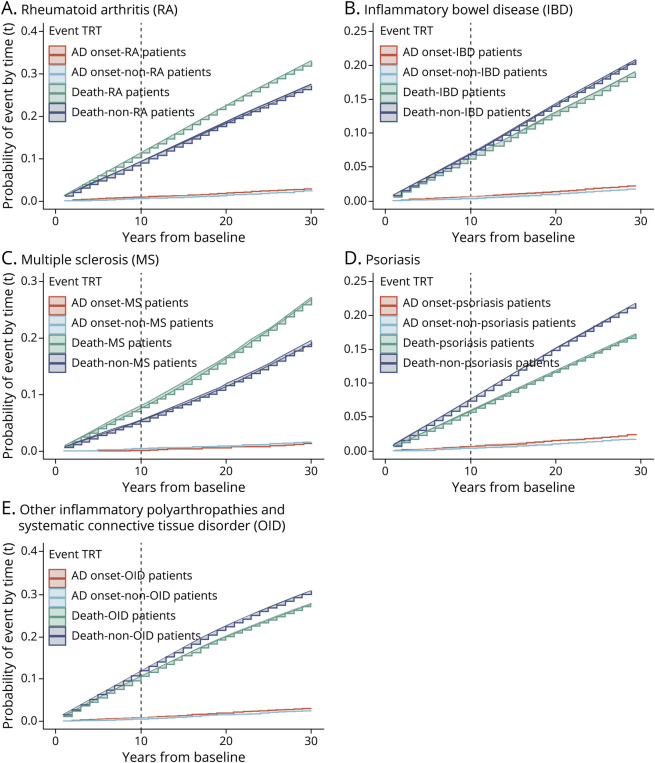
Cumulative Incidence Function Curves for Dementia Onset and for Death Using the Clinical Practice Research Datalink AD = Alzheimer disease.

Cox regression models with different covariate adjustments showed consistent findings for each of the disease relationships with AD. The overall incidence of AD was significantly higher among patients with IBD (HR 1.17; 95% CI 1.15–1.19; *p* = 2.1 × 10^−4^), OID (HR 1.13; 95% CI 1.12–1.14; *p* = 8.6 × 10^−5^), psoriasis (HR 1.13; 95% CI 1.10–1.16; *p* = 2.6 × 10^−4^), RA (HR 1.08; 95% CI 1.06–1.11; *p* = 4.0 × 10^−4^), and MS (HR 1.06; 95% CI 1.04–1.07; *p* = 2.8 × 10^−4^) compared with the corresponding nonexposed group (model 3 in Table 2). These associations also were consistent with the analysis adjusted for propensity score. Effect sizes were similar in sensitivity analyses involving further restrictions of sampling: (1) restricting the analyses with inflammatory disease diagnosis from 2004 onward, (2) using linkage-eligible patients, (3) extending the outcome from AD to recording of any dementia diagnosis, (4) using patients with both an AD diagnosis in CPRD and HES/ONS plus at least 1 dementia drug prescription in CPRD, (5) removing AD cases that developed within 2 years of inflammatory disease diagnosis, and (6) removing AD cases that developed within 3 years of inflammatory disease diagnosis (eMethods, links.lww.com/WNL/C487 and eTables 9 and 10, links.lww.com/WNL/C486). Similar associations were also found in sensitivity analyses using dementia as the outcome with a slightly higher HR among patients with IBD (HR 1.22, 95% CI 1.20–1.23, *p* = 1.7 × 10^−5^). In the time-dependent CIF analysis, the 10-year risk of developing AD in the patients with RA was 1.12% (95% CI 1.06%–1.19%) compared with 1.07% (95% CI 1.02%–1.15%) among the non-RA participants (Figure 2B and eFigure 3) and 1.09% (95% CI 1.03%–1.14%) among the patients with MS compared with 1.02% (95% CI 0.07%–1.07%) among the non-MS participants (Figure 2D and eFigure 3). The estimated 10-year risk of developing AD was also higher in patients with IBD, psoriasis, and OID compared with participants without these inflammatory diseases, whereas the 10-year risk of death was lower in patients with IBD, psoriasis, or OID (Figure 2, A, C, E, F and eFigure 3). In the sensitivity analyses with dementia as the outcome, the 10-year risk of developing dementia in patients with one of the inflammatory disease diagnoses was consistent with the findings in the analyses of AD, whereas the 10-year risk of developing dementia was higher in patients with RA (3.76%; 95% CI 3.67%–3.85%) and MS (2.45%; 95% CI 2.37%–2.52%) (eFigures 4 and 5).

**Table 2:**
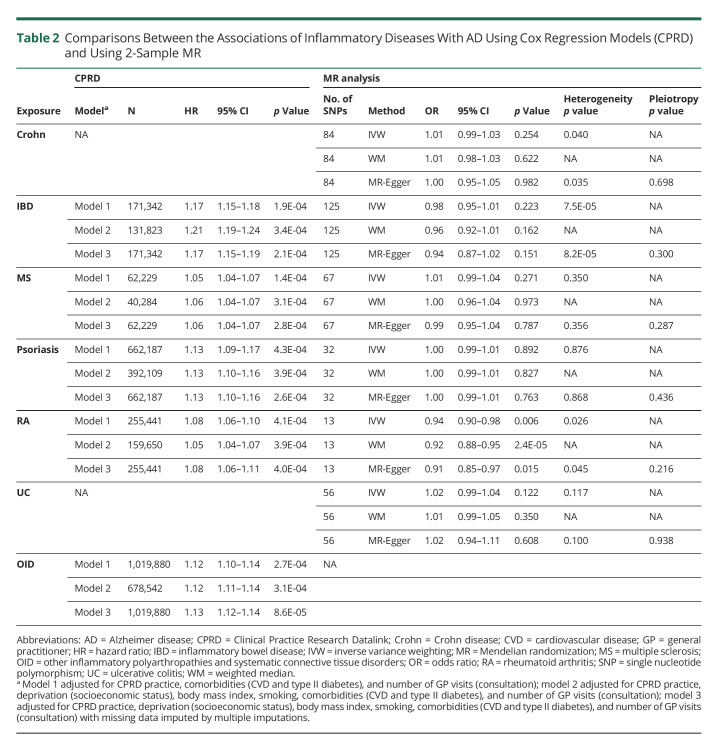
Comparisons Between the Associations of Inflammatory Diseases With AD Using Cox Regression Models (CPRD) and Using 2-Sample MR

### MR (Inflammatory Diseases and AD/Dementia)

Using the 2-sample MR approach, genetically predicted liability to inflammatory diseases was not associated with a higher risk of AD or dementia (Table 2 and eTable 11, links.lww.com/WNL/C486). After accounting for multiple comparisons, each doubling in odds of genetically predicted RA was associated with a lower risk of AD (OR_IVW_ 0.96; 95% CI 0.93–0.99; *p*_IVW_ = 0.006) and lower risk of dementia (OR_IVW_ 0.95; 95% CI 0.94–0.97; *p*_IVW_ = 3.8 × 10^−8^). However, this relationship was not sustained after excluding genetic instruments on chromosome 6 (RA→AD: OR_IVW_ 1.02; 95% CI 0.97–1.08; *p*_IVW_ = 0.44; RA→dementia: OR_IVW_ 0.99; 95% CI 0.98–1.01; *p*_IVW_ = 0.73).

### MR (Circulating Inflammatory Biomarker and AD)

**Figure 3:**
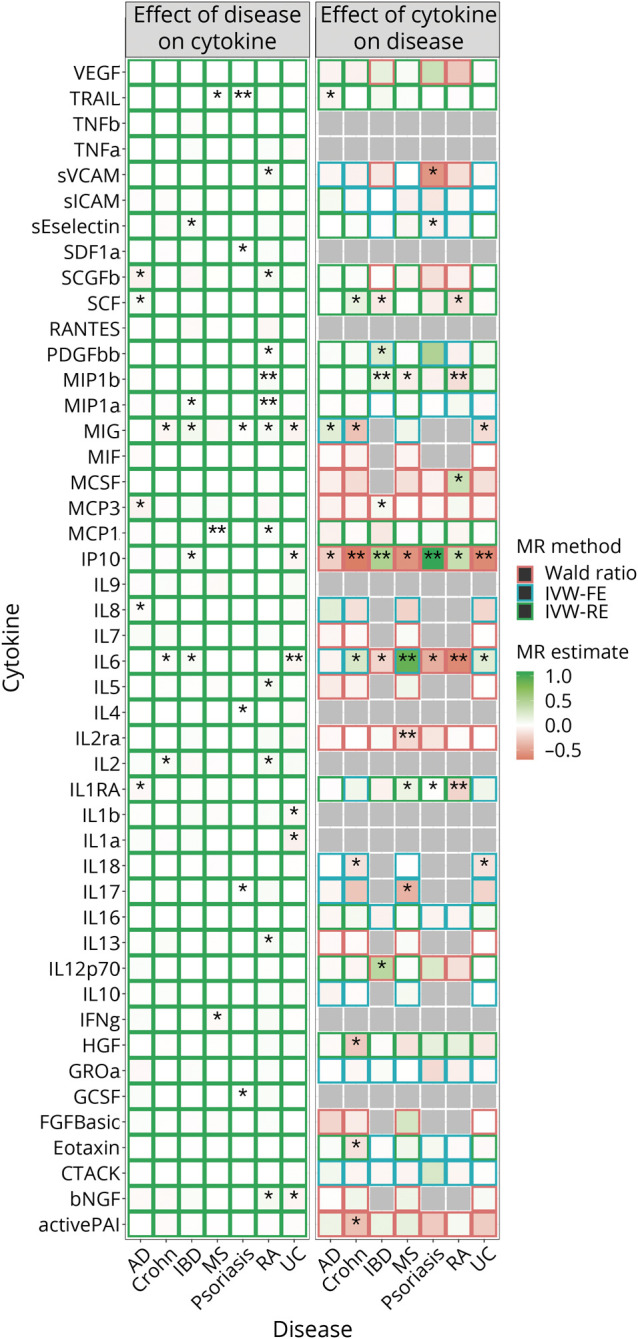
Bidirectional MR Analysis for the Associations Between Circulating Inflammatory Biomarker Levels and Diseases of Interest MR estimates were obtained from the Wald ratio method (N_SNP_ = 1), from the inverse variance weighted (IVW) fixed-effects model (IVW-FE, N_SNP_ = 2 or 3), or from the IVW random-effects model (IVW-RE, N_SNP_ > 3). *p < 0.05 and **p < 0.05/533. AD = Alzheimer disease; Crohn = Crohn disease; IBD = inflammatory bowel disease; MR = Mendelian randomization; MS = multiple sclerosis; RA = rheumatoid arthritis; UC = ulcerative colitis.

**Figure 4:**
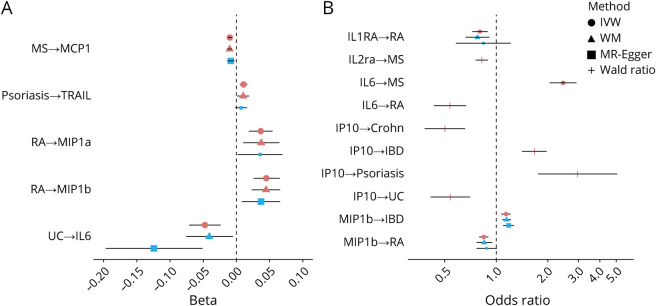
Bidirectional MR Analysis for the Associations Between Circulating Inflammatory Biomarker Levels and Diseases of Interest (*p*_Wald_ < 0.05/533 or *p*_IVW_ < 0.05/533) MR estimates were obtained from the Wald ratio method (N_SNP_ = 1), from the inverse variance weighted (IVW) fixed-effects model (IVW-FE, N_SNP_ = 2 or 3), or from the IVW random-effects model (IVW-RE, N_SNP_ > 3). Red symbol indicates *p* < 0.05/695; larger blue symbol indicates 95% CI and does not include the null value. AD = Alzheimer disease; Crohn = Crohn disease; IBD = inflammatory bowel disease; IVW = inverse variance weighting; MR = Mendelian randomization; MS = multiple sclerosis; RA = rheumatoid arthritis; UC = ulcerative colitis; WM = weighted median.

Given the well-established link between inflammation and AD,^25^ we further performed bidirectional MR analyses for circulating inflammatory biomarker levels and the diseases of interest to identify inflammatory biomarkers that are a common cause or the consequence of inflammatory disease and AD. Figure 3 presents the results from the Wald ratio or IVW, and eTables 12 and 13 (links.lww.com/WNL/C486) present the sensitivity methods. After accounting for multiple comparisons, each doubling in odds of MS was associated with a lower circulating level of MCP1 (coding gene: *CCL2*; MS→MCP1: N_SNP_ = 67; β_IVW_ = −0.01; 95% CI −0.009 to −0.005; *p*_IVW_ = 2.0 × 10^−10^). Each doubling in odds of RA was associated with a higher circulating level of MIP1a (*CCL3*; N_SNP_ = 13; β_IVW_ = 0.03; 95% CI 0.013–0.038; *p*_IVW_ = 5.1 × 10^−5^) and MIP1b (*CCL4*; N_SNP_ = 12; β_IVW_ = 0.03; 95% CI 0.018–0.045; *p*_IVW_ = 5.7 × 10^−6^). Each doubling in odds of psoriasis was associated with a higher circulating level of TRAIL (*TNFSF10*; N_SNP_ = 31; β_IVW_ = 0.01; 95% CI 0.004–0.011; *p*_IVW_ = 6.0 × 10^−5^). MR estimates from the weighted median and MR-Egger regression showed consistent directions (Figure 4), and MR-Egger did not suggest horizontal pleiotropy. Genetically predicted AD was not associated with circulating levels of any biomarkers tested.

Among the 46 biomarkers investigated, 35 had at least 1 genetic instrument for MR analysis. After accounting for multiple comparisons, each SD higher circulating MIP1b (*CCL4*) level was associated with a higher risk of IBD (N_SNP_ = 4; OR_IVW_ 1.14; 95% CI 1.07–1.21; *p*_IVW_ = 2.4 × 10^−5^), but lower risk of RA (N_SNP_ = 4; OR_IVW_ 0.84; 95% CI 0.79–0.90; *p*_IVW_ = 9.4 × 10^−8^). Each SD higher circulating IL1RA (*IL1RN*) level was associated with a lower risk of RA (N_SNP_ = 4; OR_IVW_ 0.80; 95% CI 0.72–0.90; *p*_IVW_ = 3.3 × 10^−5^). MR estimates from the weighted median and MR-Egger regression showed consistent direction (Figure 4), and MR-Egger did not suggest horizontal pleiotropy. We identified 7 more significant causal associations between circulating inflammatory biomarkers and inflammatory diseases after Bonferroni correction; however, less than 3 genetic instruments were available for these biomarkers. Thus, sensitivity methods were not performed. Particularly, genetically predicted circulating levels of interleukin (IL) 6 (*IL6*) and IP10 (*CXCL10*) were associated with more than 1 inflammatory disease. Each SD higher circulating IL6 (*IL6*) level was associated with higher risk of MS (N_SNP_ = 2; OR_IVW_ 2.45; 95% CI 2.05–2.93; *p*_IVW_ = 1.3 × 10^−22^), but lower risk of RA (N_SNP_ = 1; OR_Wald_ 0.54; 95% CI 0.43–0.67; *p*_Wald_ = 1.3 × 10^−8^). Each SD higher circulating levels of IP10 (N_SNP_ = 1) was associated with higher risk of psoriasis and IBD (IP10→psoriasis: OR_Wald_ 2.97; 95% CI 1.74–5.05; *p*_Wald_ = 6.3 × 10^−5^; IP10→IBD: OR_Wald_ 1.67; 95% CI 1.42–1.97; *p*_Wald_ = 1.3 × 10^−9^), but lower risk of Crohn disease and UC (IP10→Crohn: OR_Wald_ 0.50; 95% CI 0.38–0.66; *p*_Wald_ = 5.7 × 10^−7^; IP10→UC: OR_Wald_ 0.54; 95% CI 0.41–0.70; *p*_Wald_ = 5.4 × 10^−6^). We also found a reverse association between the circulating level of IL2ra (*IL2RA*) and the risk of MS (N_SNP_ = 1; OR_Wald_ 0.82; 95% CI 0.76–0.89; *p*_Wald_ = 3.2 × 10^−6^). The observed effect sizes for all these associations were larger than the minimum effect detectable at 85% power (eTable 14, links.lww.com/WNL/C486). However, most of the null findings in our analyses had an observed effect size smaller than the minimum detectable effect (eTable 14).

From the above MR analyses for circulating inflammatory biomarkers and inflammatory diseases, we found a bidirectional relationship between circulating MIP1b (*CCL4*) level and risk of RA. MR-Steiger suggested that both directions were valid; however, the RA→MIP1b association was less susceptible to measurement error (eTable 15, links.lww.com/WNL/C486). eFigure 6 (links.lww.com/WNL/C487) demonstrates that the MIP1b→RA association was driven by a single SNP (*p*_rs11574435-RA_ = 0.004), and the association did not sustain after exclusion of this SNP. The RA→MIP1b association remained nominally significant after excluding 2 SNPs nominally associated with MIP1b (*p*_IVW_ = 0.004).

Genetically predicted circulating inflammatory biomarker levels were not associated with the risk of AD after accounting for multiple comparisons. Nevertheless, the top signal identified for AD (circulating MIG [*CXCL9*] level [N_SNP_ = 2; OR_IVW_ 1.23; 95% CI 1.06–1.42; *p*_IVW_ = 0.007]) also was a suggestive causal biomarker for Crohn disease (N_SNP_ = 2; OR_IVW_ 0.73; 95% CI −0.62 to 0.86; *p*_IVW_ = 1.3 × 10^−4^). The observed effect sizes for these associations were larger than the minimum effect detectable at 85% power (eTable 14, links.lww.com/WNL/C486). However, colocalization did not suggest a common causal SNP for AD and Crohn disease within the genomic region (±50 kb from the MIG coding gene *CXCL9*) (posterior probability [PP.H4] = 0.001; eFigure 7, links.lww.com/WNL/C487). A common causal SNP was also unlikely for MIG and AD (PP.H4 = 0.03), although possible for MIG and Crohn disease (PP.H4 = 0.74; eFigure 8). Colocalization suggested neither a common causal SNP for IP10 (*CXCL10*) and AD (PP.H4 = 0.09), nor for IP10 (*CXCL10*) and Crohn disease (PP.H4 = 0.09; eFigure 9).

## Discussion

In this study, we performed real-world observational analysis and 2-sample MR to investigate the relationship between inflammatory diseases and AD. Consistent with previous observational studies,^4,5,26^ our real-world observational analysis based on CPRD data showed that inflammatory diseases were associated with a higher risk of AD. However, these associations were not supported by 2-sample MR analysis, suggesting that confounding factors might be driving the observed association between inflammatory diseases and AD. In MR analysis, we found that several biomarkers are likely to be affected by inflammatory diseases. Moreover, we found evidence for a potential causal role of MIG in Crohn disease. However, consistent with the results of the observational study, we did not find an inflammatory biomarker that could explain the association of inflammatory diseases and AD.

The role of inflammation in AD progression is supported by the identification of innate immune genes in genetic studies on AD, such as *CD33* and *TREM2*,^19,27^ and the emerging understanding that microglia, which contributes to the regulation of immune response in the brain, likely is central to the pathogenesis of AD.^28^ Chronic inflammation may drive neuroinflammatory changes and chronic activation of microglia, leading to oxidative stress and enhanced deposition of toxic proteins in AD.^1^ Chronic inflammation also increases the risk of thromboembolic events and ischemic stroke, contributing to the development of vascular dementia, which leads to stepwise instead of gradual cognitive decline.^29^ However, it is also possible that the observational associations between inflammatory diseases and AD are due to common molecular mechanisms.

Our findings for the relationships between circulating inflammatory biomarkers and AD were not entirely consistent with other recent studies with similar designs.^30,31^ Both Yeung et al. and Pagoni et al. used genetic associations of 41 biomarkers from the previous Finnish GWAS, which had adjusted for BMI.^30-32^ Several studies have reported a causal effect of obesity or higher BMI on inflammatory biomarker levels,^33,34^ but BMI was not a causal factor of AD or dementia.^35,36^ Using BMI-adjusted genetic associations of biomarkers could result in collider bias in 2-sample MR because of unmeasured confounding.^37^ In our study, we updated the Finnish GWAS by including 5 additional biomarkers (activePAI, IL1a, sEselectin, sICAM, and sVCAM) and excluding BMI from the genetic model. We also took advantage of 2 additional GWASs to increase the sample size of 20 biomarkers (the maximum sample size was increased from 8,293 to 13,365).^16^

Our MR findings suggested a link between MIG and higher risk of AD, but lower risk of Crohn disease. MIG belongs to the CXC chemokine family and is also known as chemokine (C-X-C motif) ligand 9 (CXCL9). Chemokines have been found to involve in both neuroinflammatory and neurodegenerative processes and play a role in the development of Aβ plaques and neurofibrillary tangles, 2 pathologic hallmarks of AD.^38^ However, colocalization in our study only supported common causal SNPs for MIG and Crohn disease, indicating that the association between MIG and AD may be confounded by linkage disequilibrium. We previously found that circulating MIG causally affects the IP10 level.^16^ MIG (*CXCL9*), IP10 (*CXCL10*), and IP9 (*CXCL11*) share the same receptor, C-X-C chemokine receptor 3 (CXCR3),^38^ but colocalization in our study did not support common causal SNPs for IP10 and Crohn disease. This may suggest a more prominent role of MIG in Crohn disease. *CXCL9* polymorphisms were found associated with Crohn disease, although only in pediatric patients.^39,40^ In addition, a recent study also identified a major role of MIG (*CXCL9*) in age-related chronic inflammation.^41^ Taken together, we did not find strong evidence supporting common inflammatory biomarkers affecting both risk of AD and inflammatory diseases. Our findings suggest that neuroinflammatory processes that are responsible for AD may be distinct from the mechanisms underlying inflammatory diseases. Nevertheless, the role of inflammatory biomarkers not being investigated in this study warrant further exploration.

There are limitations to our study. First, the observational analysis relies on the quality of data recording in the electronic health records. Some chronic autoimmune inflammatory diseases (e.g., RA, psoriasis) could be under-recorded, confounding estimates of their contributions to the risk of AD. The use of CPRD Medcodes also may provide a poor estimate of the proportion of patients with AD because nonspecific Medcodes are often used in primary care for suspected dementia and these can be misapplied.^42^ In addition, the diagnosis of AD was not confirmed by CSF biomarkers such as Aβ and tau protein. On the other hand, one systematic review assessed the validity of dementia recording in EHR and reported high false-positive rates of dementia recordings.^43^ However, recordings of AD diagnosis have improved in recent years, ^44^ and we used the linkage data set, which may provide additional patient records of AD diagnosis. Second, genetic factors explain a small proportion of the variance in the incidence of some inflammatory diseases.^45,46^ Thus, a lack of association between genetically predicted inflammatory diseases and risk of AD in our MR analysis may not rule out the contribution of inflammatory diseases to AD pathophysiology. Third, genetic associations for biomarkers were based on relatively small GWAS, which may raise the issue of lack of statistical power. Nevertheless, the observed effect sizes for our findings were larger than the minimum effect detectable at 85% power. Fourth, univariate MR analyses may not represent the direct effect of a specific biomarker on the disease outcomes because effects of one biomarker may be mediated by others in the complex biomarker network. Fifth, we primarily investigated peripheral inflammatory diseases; thus, our findings may not be able to inform on inflammation related to the CNS. In addition, circulating inflammatory biomarkers may not be relevant to neurologic diseases. However, it has been reported that some biomarkers can pass through blood-brain barrier, including IL1RA in our findings,^47^ and others can affect the integrity of blood-brain barrier, for example, MCP1.^48^ Monocytes can cross compromised blood-brain barrier and generate monocyte-derived macrophages.^49^ This may further suggest the synergistic effect of multiple biomarkers. Last but not least, all our analyses were based on individuals of European ancestry; generalization to other population requires further investigation.

In this study, we have integrated evidence from real-world observational analyses based on large sample size with a 2-sample MR analysis, which is less susceptible to confounding and reverse causation, to investigate the associations between chronic autoimmune inflammatory diseases and risk of AD and dementia. Our findings suggested that these associations were noncausal, but the factors confounding the observation have yet to be unraveled. Moreover, although some circulating inflammatory biomarkers were associated with the inflammatory diseases, evidence was weak that they affected both risk of AD and inflammatory diseases.

## Glossary

Aβ: β-amyloid
AD: Alzheimer disease
BMI: body mass index
CIF: cumulative incidence function
CPRD: Clinical Practice Research Datalink
EHR: electronic health record
GP: general practitioner
GWAS: genome-wide association study
HES: Hospital Episode Statistics
HR: hazard ratio
IBD: inflammatory bowel disease
IGAP: International Genomics of Alzheimer's Project
IL: interleukin
IMD: index of multiple deprivation
IPTW: inverse propensity score treatment weighting
IVW: inverse-variance weighting
MIG: monokine induced by gamma interferon
MR: Mendelian randomization
MS: multiple sclerosis
OID: other inflammatory polyarthropathies and systematic connective tissue disorders
ONS: Office of National Statistics
OR: odds ratio
PP.H4: posterior probability
RA: rheumatoid arthritis
SNP: single nucleotide polymorphism
UC: ulcerative colitis
